# Supplementary material for: Why Did ZIKV Perinatal Outcomes Differ in Distinct Regions of Brazil? An Exploratory Study of Two Cohorts
Source: Viruses. 2021 Apr 23;13(5):736. doi: 10.3390/v13050736 (PMC8146858; doi:10.3390/v13050736)

viruses MDPI

Supplemental Material

Supplemental Figure S1. DENV antibody avidity, stratified by normal and adverse gestational outcomes.

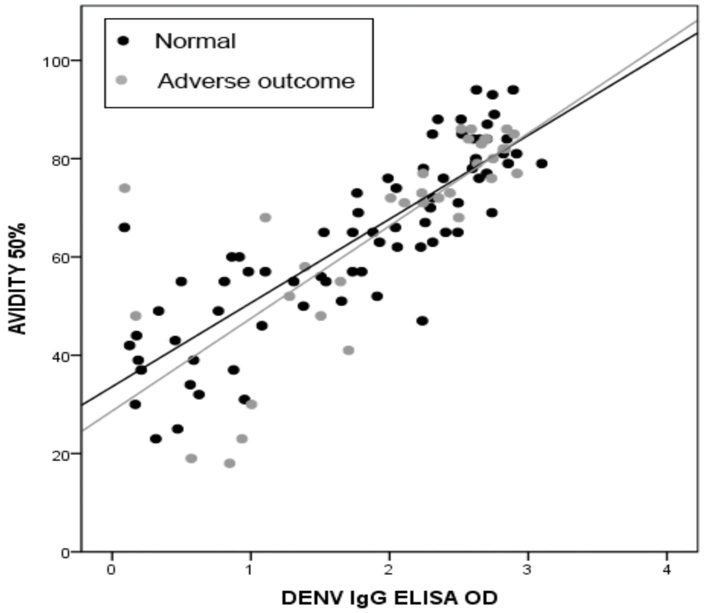

Supplemental Figure S2. DENV antibody avidity in pregnant women infected with ZIKV, stratified by normal or adverse gestational outcomes.

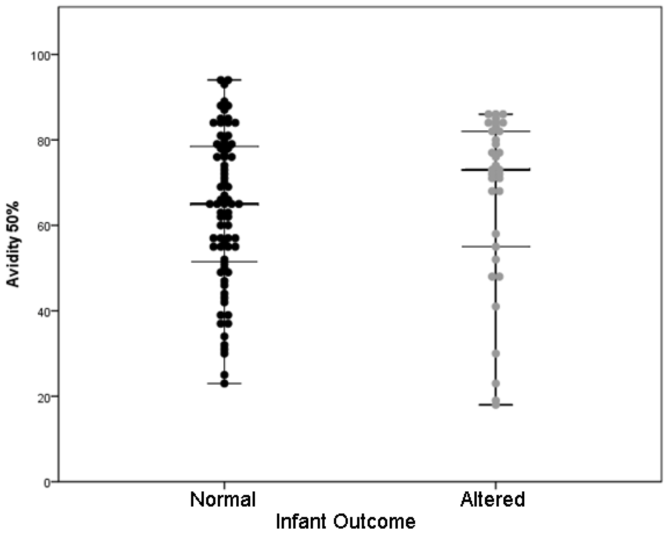

Supplement: Supplementary file 1 [file viruses-13-00736-s001.zip › viruses-1152865-supplementary.pdf]
